# The impact of wearable sports equipment on college students’ physical exercise persistence: a mediated model of physical exercise motivation moderated by social support

**DOI:** 10.3389/fspor.2025.1691032

**Published:** 2025-10-17

**Authors:** Yufeng Han, Sen Wang, Zhiwen Zhang, Junli Fan

**Affiliations:** ^1^ College of Physical Education, Jeonbuk National University, Jeonju, Republic of Korea; ^2^ Zhengzhou Zhengdongxinqu Foreign Language School, Zhengzhou, Henan, China; ^3^ College of Economics and Management, Chongqing Vocational College of Transportation, Chongqing, China

**Keywords:** wearable sports equipment, physical exercise persistence, exercise motivation, social support, mediation-moderation

## Abstract

**Background:**

Under the strategic framework of “Healthy China,” the issue of insufficient exercise adherence among college students has become increasingly prominent. As an emerging intervention tool, wearable sports equipment (WSE) holds potential in addressing this issue, yet its effectiveness may be influenced by exercise motivation and social support. Existing studies have primarily focused on the independent effects of technological interventions, with limited exploration of the psychosocial mechanisms involved.

**Objective:**

Drawing upon Self-Determination Theory and Social Support Theory, this study constructs a mediated model of exercise motivation to examine the mechanisms through which WSE influences exercise adherence among college students, with a particular focus on the mediating role of exercise motivation and the moderating effect of social support.

**Methods:**

A cross-sectional survey was conducted using stratified cluster sampling among 1,286 students from six universities across China. Core variables were measured using the Perceived Use of Wearable Equipment Scale, Exercise Adherence Scale, Exercise Motivation Scale, and Social Support Scale. Model 59 of the SPSS PROCESS macro was employed for data analysis.

**Results:**

WSE use significantly predicted exercise adherence (*β* = 0.143, *p* < 0.001), with exercise motivation partially mediating this relationship (accounting for 27.3% of the total effect). Social support exhibited a dual moderating effect: it strengthened the direct effect of WSE on exercise adherence (*β* = 0.204 under high support vs. *β* = 0.082 under low support), but weakened the effect of WSE on exercise motivation (non-significant under high support, *β* = 0.219 under low support), as well as the indirect effect of WSE on exercise adherence via motivation (*β* = 0.116 under high support vs. *β* = 0.463 under low support).

**Conclusion:**

WSE impacts exercise behavior through the synergistic interplay of exercise motivation and social support. Intervention strategies should be tailored according to individuals’ levels of social support—those with low support should focus on strengthening motivational internalization, while those with high support may benefit more from the direct reinforcement of technological feedback. The findings provide a theoretical basis for optimizing health promotion strategies in higher education institutions.

## Introduction

In recent years, the Chinese government has increasingly emphasized the importance of public health, introducing a series of policies aimed at encouraging regular physical activity across the population. The 2016 “*Healthy China 2030” Planning Outline* clearly stated that “public health is the ultimate goal of building a healthy China,” with one of its core objectives being to “promote the integration of nationwide fitness and overall health” (1). Subsequently, the General Administration of Sport, in collaboration with the Ministry of Education and other departments, issued the *Youth Physical Activity Promotion Plan*, which highlighted the use of technology to increase exercise participation among young people, including college students (2). In 2021, the “National Fitness Plan (2021–2025)” further stressed the need to “apply modern technologies such as wearable sports equipment to create innovative health service models,” specifically to address the issue of weak physical exercise persistence among young individuals (3). These policy trends reflect a growing national strategy to promote healthy lifestyles by incorporating wearable sports equipment—such as smart wristbands and fitness watches—into interventions aimed at improving exercise habits among college students. Furthermore, recent meta-analytic evidence has demonstrated the efficacy of nature-based social prescriptions in improving mental health outcomes, which aligns with the national strategy of promoting holistic health through multi-faceted interventions (4). Additionally, studies have highlighted that digital platforms and virtual fitness technologies can significantly enhance motivation and knowledge development among university students, suggesting a promising avenue for integrating technology with traditional health promotion methods (5).

At the same time, there is growing concern over the physical health of the college student population. According to the 2020 *National Report on Students' Physical Fitness and Health*, only 23.8% of Chinese college students met the national fitness standards, with low exercise motivation and lack of social support being identified as key factors linked to poor physical exercise persistence (6). While traditional campus-based physical education policies (such as integrated in- and out-of-class physical activity programs) may temporarily increase activity levels, research suggests their long-term impact is limited (7, 8). Against this backdrop, wearable sports equipment has been recognized for its potential to improve individuals' self-regulation in exercise through features such as real-time feedback and goal setting (9, 10). However, current policies have paid insufficient attention to how technological interventions interact with psychological and social mechanisms—particularly exercise motivation and social support. For example, although the *Basic Standards for Physical Education in Higher Education Institutions* require universities to “establish student physical health records,” they provide no clear guidance on how to integrate data from wearable equipments with psychological support to optimize intervention outcomes (11).

International experience also supports the value of combining technology with social support. The World Health Organization (WHO), in its *Global Action Plan on Physical Activity 2018–2030*, emphasizes the importance of enhancing exercise adherence through a dual pathway that integrates “digital tools and social networks”. Similarly, the American College of Sports Medicine (ACSM), in its position statement, noted that wearable sports equipment should be used in tandem with support from peers or mentors in order to bring about sustained improvements in physical activity behavior (12–14). Based on both China's policy priorities and international insights, this study focuses on the moderating role of social support in interventions using wearable sports equipment, aiming to bridge the gap between policy and practice and to provide a theoretical foundation for sports and health management in colleges and universities.

## The relationship between wearable sports equipment and physical exercise persistence among college students

In recent years, wearable sports equipment—such as smart wristbands and fitness watches—has emerged as a valuable tool in promoting physical exercise persistence, thanks to its functions in data tracking, real-time feedback, and behavioral reinforcement (15, 16). Among college students, who typically show relatively low levels of exercise adherence (6), physical activity behavior is shaped by a range of factors including exercise motivation, self-regulation ability, and the surrounding social environment (17). Research has shown that wearable sports equipment can enhance physical exercise persistence through several psychological mechanisms, such as goal setting, self-monitoring, and social comparison (18–20). However, the effectiveness of such interventions appears to vary significantly between individuals. This variation may be explained, at least in part, by the moderating effects of social support and the mediating role of exercise motivation (21–23). Therefore, examining how wearable sports equipment supports exercise behavior among college students through the mediating pathway of exercise motivation, while also considering the moderating role of social support, is of both theoretical relevance and practical value.

The key strength of wearable sports equipment lies in its ability to provide real-time data—such as step count, heart rate, and calorie consumption—which helps users develop a more objective understanding of their physical activity (24–26). According to Social Cognitive Theory (27), the stronger a person's ability to monitor their own behavior, the higher their sense of self-efficacy tends to be, and the more likely they are to maintain a consistent exercise routine. For example, an experimental study involving college students found that participants who used smart wristbands significantly increased their daily step count over an eight-week intervention period (*p* < 0.01), along with improvements in self-regulation ability (28). In addition, features such as achievement systems (e.g., badges, leaderboards) can reinforce behavior through operant conditioning mechanisms, encouraging users to remain engaged in physical activity (29–31). That said, relying solely on technological feedback may give rise to what is known as “data fatigue”—a drop in user engagement due to repeated exposure to similar forms of motivation over time (32). This suggests that the intervention effect of wearable sports equipment is not merely determined by its technical functions, but is also shaped by individual psychological factors (such as exercise motivation) and external conditions (such as social support). Moreover, during the COVID-19 pandemic, the role of technology in mitigating the negative impacts of isolation on physical and psychological health has been widely acknowledged. For instance, virtual reality fitness was found to mediate the relationship between preventive measures and overall wellbeing, highlighting the potential of technology-assisted interventions in crisis contexts (33).

## The relationship between wearable sports equipment, exercise motivation, and physical exercise persistence among college students

The growing popularity of wearable sports equipment—including smart bands and fitness watches—has opened new possibilities for studying college students' exercise behaviors from a technology-enhanced perspective. Research indicates that these equipments, through features like real-time activity tracking (e.g., step count, heart rate, calorie burn) and immediate feedback, contribute meaningfully to users' ability to self-regulate their physical activity (24–26). However, significant differences in the outcomes of equipment usage have been observed between individuals. One possible explanation for this variation is the mediating role of exercise motivation, a core psychological factor (17). According to Self-Determination Theory (SDT), physical exercise persistence is not only linked to external interventions but is also closely related to how deeply an individual internalizes their exercise motivation (34, 35). In this sense, understanding how wearable sports equipment contributes to changes in physical exercise persistence through its relationship with exercise motivation provides critical insight into the underlying psychological mechanisms of technology-based exercise interventions.

A number of empirical studies have shown that wearable sports equipment can play a role in enhancing exercise motivation. For instance, Fritz et al. (20) found that goal achievement feedback provided by these equipments strengthens users' sense of competence, which in turn contributes to higher levels of intrinsic motivation. This finding echoes Bandura's (27) self-efficacy theory, which suggests that when individuals are able to confirm their physical capabilities through objective data, they are more likely to stay engaged in regular physical activity. Among Chinese college students, Tam (28) conducted an intervention study and observed that participants using smart wristbands not only showed a notable increase in daily step count after eight weeks, but also reported higher levels of exercise enjoyment—a key indicator of intrinsic motivation—compared to the control group. These results suggest that wearable sports equipment may support motivation internalization by satisfying the three basic psychological needs outlined in Self-Determination Theory (SDT): autonomy (through self-set goals), competence (through data-based validation), and relatedness (through social features) (36).

That said, it's important to recognize that the relationship between equipment usage and exercise motivation is not necessarily linear. In a longitudinal study, Peng et al. (37) found that while wearable sports equipment initially boosts exercise frequency via external motivation (such as earning virtual badges), long-term physical exercise persistence is more closely related to intrinsic motivation. This highlights a possible “motivation shift” over time—users may rely on external rewards early on, but sustained engagement tends to require internal motivational support. Moreover, overreliance on equipment-based feedback may lead to what has been called “data fatigue” (38, 39), where users grow tired of repetitive motivational patterns and gradually lose interest. This further underscores the need to understand how motivation changes over time, especially when designing technology-based interventions aimed at supporting consistent physical activity. Recent evidence also suggests that coping behaviors play a crucial mediating role in alleviating the effects of a pandemic on students' physical and psychological health, which further underscores the importance of psychosocial factors in health intervention models (40). Furthermore, social support has been identified as a key moderator that enhances the effectiveness of digital interventions, particularly in promoting motivation and adherence among young adults (5, 40).

## The relationship among wearable sports equipment, exercise motivation, social support, and college students' physical exercise persistence

As the *Healthy China* strategy continues to gain traction, college students' physical activity habits have attracted growing academic attention. Wearable sports equipment—such as fitness trackers and smartwatches—have emerged as promising tools for health intervention. By offering real-time monitoring, goal-setting features, and instant feedback, these equipments provide practical technological support for improving physical exercise persistence among college students (41–44). Still, the effectiveness of these tools varies widely between individuals, prompting researchers to take a closer look at the roles of exercise motivation and social support in shaping these outcomes (17). Drawing on Self-Determination Theory (SDT) and the theory of social support, the present study proposes a mediated model in which exercise motivation serves as the psychological mechanism linking wearable sports equipment use to physical exercise persistence. In addition, the model explores how social support plays a moderating role within this process, aiming to provide both theoretical insight and practical guidance for improving physical activity interventions among university students.

Social support serves as a critical moderating factor in this model. Based on social exchange theory, support from peers, family members, or coaches can strengthen the positive outcomes related to wearable sports equipment use (45). Research has shown that users who receive in-person guidance while using such equipments demonstrate physical exercise persistence levels that are 31% higher than those who use the equipments independently (46, 47). Within the Chinese cultural context, Xu et al. (48) found that family support is closely related to enhanced exercise motivation among equipment users (48). This may be because social support offers not only emotional affirmation but also behavioral modeling, which can help users internalize the feedback provided by the equipment more effectively (22, 49). It is worth noting that different types of social support may be related to distinct outcomes. Instrumental support—such as exercising together—tends to be more directly related to physical exercise persistence, whereas emotional support—like encouragement or positive reinforcement—has a more indirect connection by reinforcing exercise motivation (50).

## Research hypotheses

Grounded in Self-Determination Theory and the theory of social support, this study proposes the following hypotheses:

H1: The use of wearable sports equipment is positively related to college students' physical exercise persistence.

H2: Exercise motivation plays a mediating role in the relationship between wearable sports equipment and physical exercise persistence.

H3: Social support moderates the relationship between wearable sports equipment and exercise motivation.

H4: Social support moderates the relationship between exercise motivation and physical exercise persistence.

H5: Social support moderates the relationship between wearable sports equipment and physical exercise persistence.

**Figure 1:**
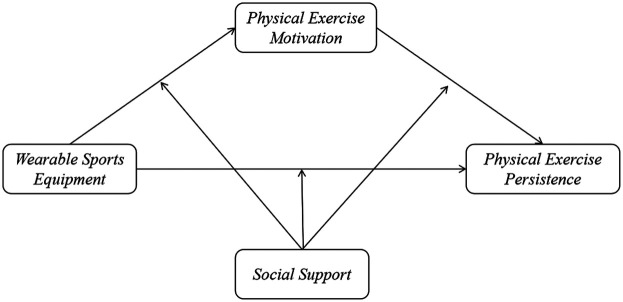
Moderated mediation model of wearable sports equipment, physical exercise persistence, physical exercise motivation and social support.

These hypotheses will be tested using a moderated mediation model (see Figure 1). The results are expected to provide insight into how technological interventions interact with psychosocial factors in shaping physical activity behavior among college students. This may offer theoretical guidance for developing tailored health promotion strategies.

## Research method

### Participants

This study adopted a stratified cluster sampling method to recruit undergraduate students from six comprehensive universities located in eastern, central, and western China. Among these universities, two were “Double First-Class” institutions and four were ordinary undergraduate universities. The inclusion criteria were as follows: (1) full-time undergraduate students; (2) aged between 18 and 24 years old; (3) no regular physical exercise over the past six months (defined as less than 150 min of moderate-intensity exercise per week); and (4) voluntarily agreed to participate and signed the informed consent form. The exclusion criteria included: (1) individuals with medical contraindications to physical activity; (2) participants currently involved in other exercise intervention programs; and (3) those who experienced difficulties using smart equipments.

A total of 1,286 valid responses were collected (46.0% male, 54.0% female), with an average age of 20.02 ± 1.43 years. Freshmen accounted for 25.0%, sophomores 25.3%, juniors 24.9%, and seniors 24.8% of the sample. In terms of academic majors, 31.3% were from humanities and social sciences, 34.0% from science and engineering, and 34.8% from medical-related disciplines.

The required minimum sample size was calculated using G*Power 3.1 software, with a medium effect size (*f*^2^ = 0.15), significance level *α* = 0.05, and statistical power (1−*β*) = 0.95. The minimum required sample size for multiple regression analysis was 1,072, indicating that the actual sample size sufficiently met the requirements for statistical analysis. Demographic characteristics of the participants are presented in Table 1. This study was approved by the Ethics Jiangxi Normal University (approval number: IRB-JXNU-PSY-20250016) and all procedures were conducted in accordance with the ethical standards outlined in the *Declaration of Helsinki.*

**Table 1:** Presents the descriptive statistics and correlations among the main variables.

Variable	*M*	SD	WSE	PEP	PEM	SS
WSE	2.979	1.111	1			
PEP	2.319	0.878	.488**	1		
PEM	2.388	0.820	.675**	.796**	1	
SS	2.424	0.847	.664**	.826**	.905**	1

Values are Pearson correlation coefficients; **p < .01 (two-tailed).

*N* = 1,072. WSE, the use of wearable sports equipment; PEP, physical exercise persistence; PEM, physical exercise motivation; SS, social support.

### Research instruments

#### Questionnaire on the use of wearable sports equipment

The measurement of wearable sports equipment usage was based on a combination of established instruments developed by Song J. et al. (51), including the Perceived Usefulness Scale of Wearable equipments, the Motivation Scale for Using Wearable equipments, and the Trust in Wearable Technology Scale. The original reliability coefficients of these scales were 0.926, 0.928, and 0.937 respectively, and all have been widely applied in previous domestic and international studies.This section of the questionnaire consisted of 34 items in total. The dimension of motivation related to wearable sports equipment usage was measured by 11 items (e.g., “Using wearable equipments encourages me to engage in daily physical activity positively”); perceived usefulness was assessed with 10 items (e.g., “Wearable equipments help me achieve my exercise goals more efficiently”); and trust in wearable technology was evaluated by 13 items (e.g., “I believe the data shown on wearable equipments is accurate”). A 5-point Likert scale was used to score the items, with higher scores indicating a higher level of engagement with wearable sports equipment. To better capture the impact of different types of WSE devices, this study introduced new metrics to differentiate WSE functionalities: device type (basic/professional/social) and core feature usage frequency (e.g., weekly data tracking usage, weekly social interaction participation). This approach will help us explore how different features influence exercise endurance and motivation. In this study, the overall instrument demonstrated strong psychometric properties (Cronbach's *α* = 0.981, *χ*^2^/df = 2.624, CFI = 0.977, TLI = 0.975, RMSEA = 0.036, SRMR = 0.033).

#### Questionnaire on physical exercise persistence

To measure physical exercise persistence, this study used the “Exercise Adherence Scale” developed by Professor Wang et al. (52). The scale divides physical exercise persistence into three dimensions, with a total of 14 items. The dimension of exercise behavior includes 4 items (e.g., “Each time I engage in physical activity, it lasts for at least one hour”); effort and investment consists of 5 items (e.g., “I actively practice new skills to improve myself”); and emotional experience contains 5 items (e.g., “I enjoy the feeling that physical activity brings to me”). Previous research has shown that the original version of this scale has good reliability and validity, and it has been widely used in China. This questionnaire uses a 5-point Likert scale, with higher scores indicating a higher level of physical exercise persistence. In the current study, this instrument demonstrated strong psychometric properties (Cronbach's *α* = 0.923, *χ*^2^/df = 2.714, CFI = 0.983, TLI = 0.980, RMSEA = 0.037, SRMR = 0.030).

### Questionnaire on exercise motivation

To assess exercise motivation, the study employed the Chinese version of the “Motives for Physical Activity Measure – Revised” (MPAM-R), translated and adapted by Chen Shanping based on the local context. The questionnaire evaluates exercise motivation across five dimensions: health, enjoyment, competence, appearance, and social interaction. Each dimension includes 3 items, making a total of 15 items. Participants rated each item using a 5-point Likert scale (from 1 = “Not at all” to 5 = “Very strongly”). During translation and validation, Chen and colleagues tested the feasibility and validity of the instrument. Item analysis showed that all items had good discrimination, internal consistency, and structural validity. The scale has been shown to be suitable for capturing exercise motivation among the target population. Higher total scores reflect stronger levels of exercise motivation. In the current study, this scale also showed good reliability and validity (Cronbach's *α* = 0.924, *χ*^2^/df = 2.750, CFI = 0.981, TLI = 0.978, RMSEA = 0.037, SRMR = 0.030).

#### Questionnaire on social support

Social support was measured using the Social Support Rating Scale originally developed by Vaux et al. (53) and later revised by Xin et al. (54). The questionnaire across three dimensions: family support, friends support and others support. This instrument contains 20 items, such as “My friends respect me.” Responses were rated on a 5-point Likert scale, ranging from 1 (“Never true”) to 5 (“Always true”). This study adds a distinction between online and offline nature of social support: offline support (such as face-to-face support from family and friends) and online support (such as virtual support from fitness app communities, social media, etc.). In this study, the Cronbach's *α* coefficient of the scale was 0.930. Higher total scores indicate a higher level of social support. The scale demonstrated good psychometric properties in the current study (Cronbach's *α* = 0.947, *χ*^2^/df = 2.270, CFI = 0.983, TLI = 0.981, RMSEA = 0.031, SRMR = 0.029).

### Data analysis

Descriptive statistics, correlation analysis, and common method bias testing were conducted using SPSS 26.0. To examine the mediated model moderated by social support, the study used PROCESS macro (Model 59) in SPSS.

## Results

### Common method bias test

The analysis involved 42 items in total, from which 9 factors were extracted. The first factor accounted for 29.972% of the total variance, well below the commonly accepted threshold of 50%, suggesting that common method bias was unlikely to pose a serious threat to the findings. The cumulative explained variance reached 67.763%, exceeding the recommended threshold of 60%, indicating that the nine extracted factors were able to represent the variability in the data reasonably well.

### Correlation analysis

As shown in Table 1, the descriptive statistics and correlation results for each variable are as follows: the mean score for wearable sports equipment usage was 2.979 (SD = 1.111), for physical exercise persistence was 2.319 (SD = 0.878), for exercise motivation was 2.388 (SD = 0.820), and for social support was 2.424 (SD = 0.847).

**Figure 2:**
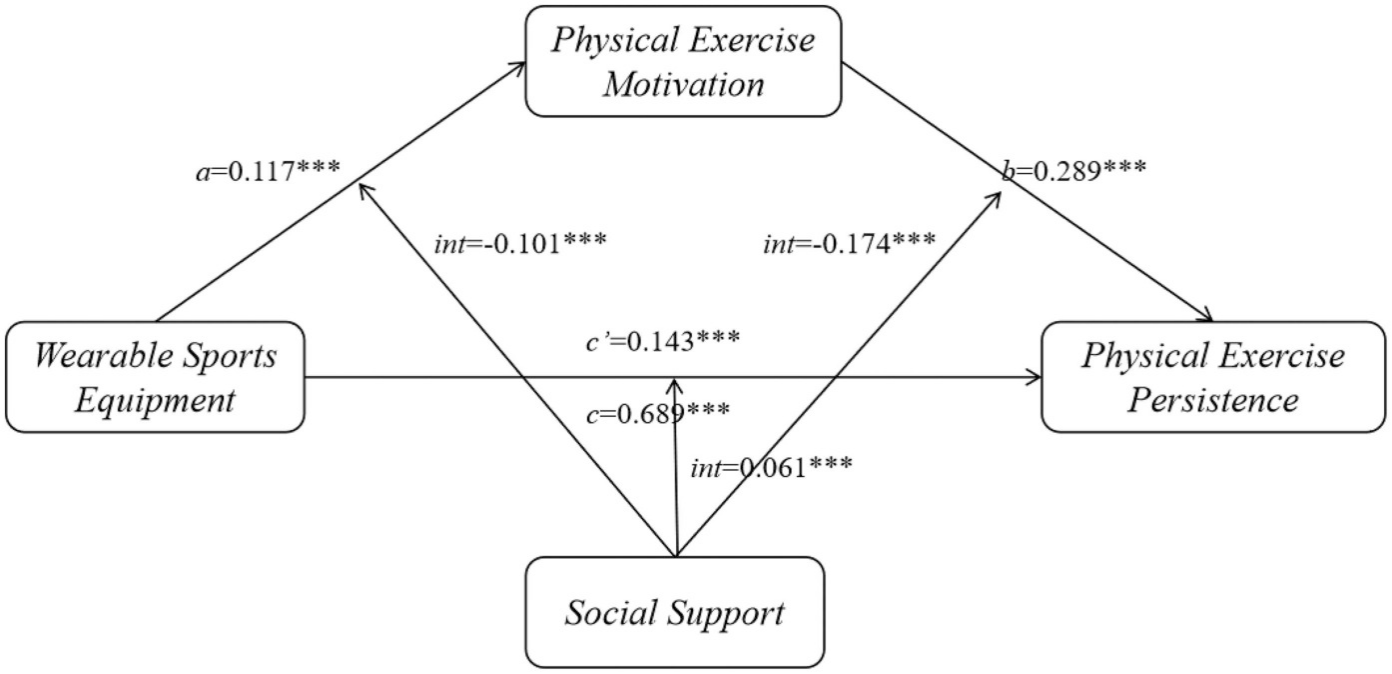
Mediated model with moderation effects.

The Pearson correlation analysis indicated that wearable sports equipment usage was positively related to physical exercise persistence (*r* = 0.488, *p* < 0.01), and also showed significant positive relations with exercise motivation (*r* = 0.675, *p* < 0.01) and social support (*r* = 0.664, *p* < 0.01) (see Figure 2). In addition, physical exercise persistence was positively related to both exercise motivation (*r* = 0.796, *p* < 0.01) and social support (*r* = 0.826, *p* < 0.01), while the relation between exercise motivation and social support was also strongly positive (*r* = 0.905, *p* < 0.01). These findings provide solid empirical groundwork for the following analysis of the mediated model.

### Test of the mediated model moderated by social support

To examine the moderated mediation effect, we used the PROCESS macro in SPSS, specifically Model 59, to analyze how social support moderates the model and how exercise motivation serves as a mediator. Details of the analysis are presented below:

As shown in Table 2, the results of the moderated mediation model indicated that wearable sports equipment usage was positively related to both physical exercise persistence (*β* = 0.143, *p* < 0.001) and exercise motivation (*β* = 0.117, *p* < 0.001). Social support was not only directly and positively related to physical exercise persistence (*β* = 0.449, *p* < 0.001) and exercise motivation (*β* = 0.781, *p* < 0.001), but also significantly moderated the relation between wearable sports equipment usage and the two outcome variables. The interaction terms were statistically significant (*β* = 0.061 and −0.101, respectively, both *p* < 0.001). Additionally, exercise motivation showed a significant positive relation with physical exercise persistence (*β* = 0.289, *p* < 0.001), and this relation was negatively moderated by social support (*β* = −0.174, *p* < 0.001).

**Table 2:** Test of the moderated mediation model between wearable sports equipment usage and physical exercise persistence.

Variable	PEP				PEM			
	*β*	SE	*t*	*p*	*β*	*SE*	*t*	*p*
WSE	0.143	0.017	8.666	<0.001	0.117	0.015	7.714	<0.001
SS	0.449	0.029	15.275	<0.001	0.781	0.016	9.717	<0.001
WSE × SS	0.061	0.016	3.883	<0.001	−0.101	0.013	−7.988	<0.001
PEM	0.289	0.030	9.738	<0.001				
PEM × SS	−0.174	0.018	−9.482	<0.001				
*N*	1,286				1,286			
*R*	0.907				0.915			
*R2*	0.823				0.837			
*F*	*F* (5, 1,280) = 188.427, p = 0.000				*F* (3, 1,282) = 197.719, p = 0.000			

*N* = 1,072. WSE, the use of wearable sports equipment; PEP, physical exercise persistence; PEM, physical exercise motivation; SS, social support.

The analysis of the moderating role of social support in the relation between wearable sports equipment usage and exercise motivation (see Table 3) revealed the following: under a low level of social support (−1 SD), wearable sports equipment usage was significantly related to increased exercise motivation [Effect = 0.219, *p* < 0.001, 95% CI (0.183, 0.255)]; at the mean level of social support, this relation remained significant but was weaker [Effect = 0.117, *p* < 0.001, 95% CI (0.088, 0.147)]; however, under a high level of social support (+1 SD), the relation was no longer statistically significant [Effect = 0.016, *p* = 0.452, 95% CI (−0.026, 0.057)]. This pattern suggests that social support significantly moderates the relation between wearable sports equipment usage and exercise motivation, and as the level of social support increases, the positive relation between equipment usage and motivation becomes weaker.

**Table 3:** Moderating effect of social support in the relation between wearable sports equipment usage and exercise motivation.

Level	Value	Effect	BootSE	*t*	*p*	BootLLCI	BootULCI
Low (−1SD)	−1.000	0.219	0.018	11.862	0.000	0.183	0.255
Mean	0.000	0.117	0.015	7.714	0.000	0.088	0.147
High (+1SD)	1.000	0.016	0.021	0.753	0.452	−0.026	0.057

BootLLCI refers to the lower bound of the 95% confidence interval obtained through bootstrapping; BootULCI refers to the upper bound of the same interval. The type of bootstrap method used is percentile bootstrap.

As shown in Table 4, the moderating effect of social support on the relation between wearable sports equipment usage and physical exercise persistence also demonstrated clear differences. At a low level of social support (−1 SD), the positive relation between equipment usage and physical exercise persistence was relatively weak but still significant [Effect = 0.082, *p* < 0.001, 95% CI (0.039, 0.126)]; at the mean level, the relation became stronger [Effect = 0.143, *p* < 0.001, 95% CI (0.111, 0.177)]; and under high social support (+1 SD), the relation was the strongest [Effect = 0.204, *p* < 0.001, 95% CI (0.158, 0.250)]. These results suggest that social support enhances the relation between wearable sports equipment usage and physical exercise persistence, with the facilitative effect of equipment usage becoming increasingly stronger as social support rises.

**Table 4:** Moderating effect of social support in the relation between wearable sports equipment usage and physical exercise persistence.

Level	Value	Effect	BootSE	*t*	*p*	BootLLCI	BootULCI
Mean−1SD	−1.000	0.082	0.022	3.717	0.000	0.039	0.126
Mean	0.000	0.143	0.017	8.665	0.000	0.111	0.177
Mean +1SD	1.000	0.204	0.024	8.701	0.000	0.158	0.250

BootLLCI refers to the lower bound of the 95% confidence interval generated by bootstrap sampling, and BootULCI refers to the upper bound. The bootstrap method used is the percentile bootstrap approach.

Results shown in Table 5 further revealed that social support significantly moderated the relation between exercise motivation and physical exercise persistence. Under low social support (−1 SD), exercise motivation was most strongly related to physical exercise persistence [Effect = 0.463, *p* < 0.001, 95% CI (0.396, 0.540)]; this relation weakened but remained significant at the mean level [Effect = 0.289, *p* < 0.001, 95% CI (0.231, 0.348)]; and under high social support (+1 SD), the relation further declined [Effect = 0.116, *p* = 0.001, 95% CI (0.046, 0.186)]. These findings indicate a clear pattern of negative moderation: as social support increases, the strength of the relation between exercise motivation and physical exercise persistence becomes progressively weaker.

**Table 5:** Moderating effect of social support in the relation between exercise motivation and physical exercise persistence.

Level	Value	Effect	BootSE	*t*	*p*	BootLLCI	BootULCI
Mean −1SD	−1.000	0.463	0.034	13.606	0.000	0.396	0.540
Mean	0.000	0.289	0.030	9.738	0.000	0.231	0.348
Mean +1SD	1.000	0.116	0.036	3.234	0.001	0.046	0.186

BootLLCI refers to the lower bound of the 95% confidence interval generated by bootstrap sampling, and BootULCI refers to the upper bound. The bootstrap method used is the percentile bootstrap approach.

**Figure 3:**
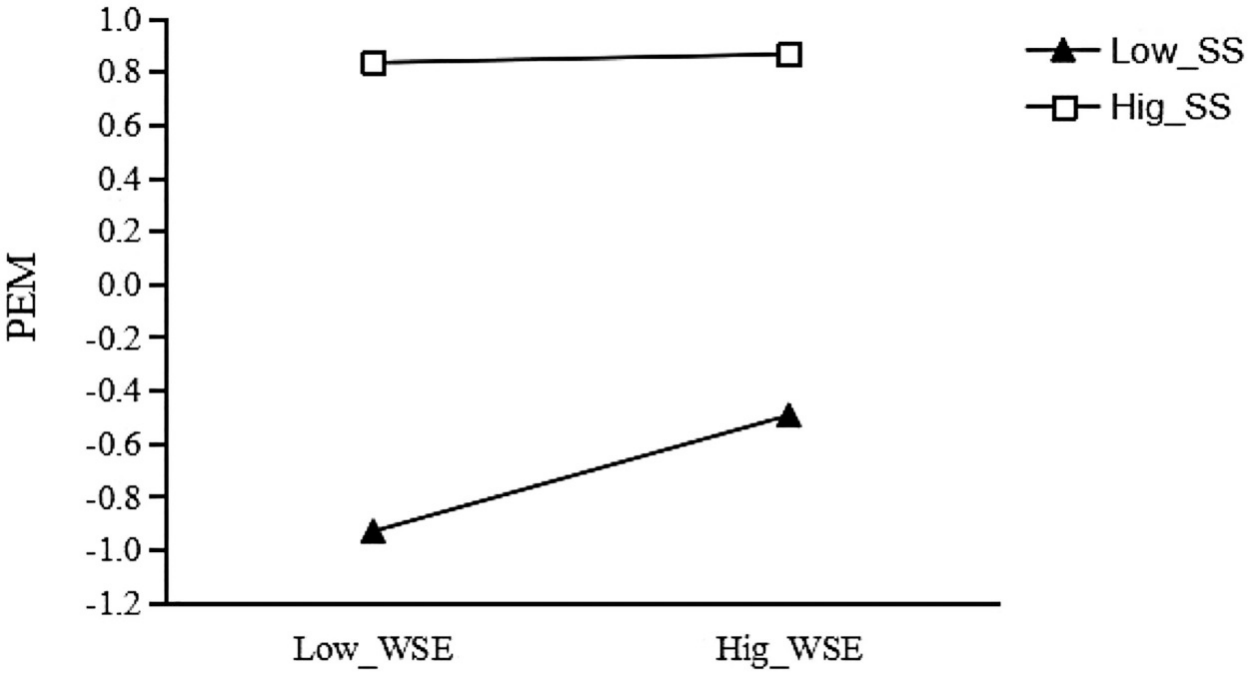
Moderating effect of social support between wearable sports equipment and exercise motivation.

For the group with low social support (see Figure 3), the effect of wearable sports equipment usage on exercise motivation was significant (*β* = 0.219, *t* = 11.862, *p* < 0.001); for each unit increase in wearable sports equipment usage, college students' exercise motivation increased by 0.219 units. However, for the group with high social support, the effect of wearable sports equipment usage on exercise motivation was not significant (*β* = 0.016, *t* = 0.753, *p* > 0.05). This suggests that social support plays a significant negative moderating role in the relationship between wearable sports equipment usage and exercise motivation.

**Figure 4:**
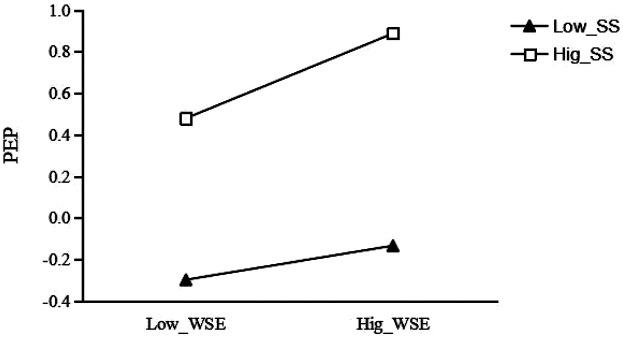
Moderating effect of social support on the relationship between wearable sports equipment usage and physical exercise persistence.

For the group with low social support (see Figure 4), the effect of wearable sports equipment usage on physical exercise persistence was significant (*β* = 0.082, *t* = 3.717, *p* < 0.001); for each unit increase in wearable sports equipment usage, college students' physical exercise persistence increased by 0.082 units. For the group with high social support, the effect of wearable sports equipment usage on physical exercise persistence was also significant (*β* = 0.204, *t* = 8.701, *p* < 0.001); for each unit increase in wearable sports equipment usage, college students' physical exercise persistence increased by 0.204 units. This indicates that social support plays a significant positive moderating role in the relationship between wearable sports equipment usage and physical exercise persistence.

**Figure 5:**
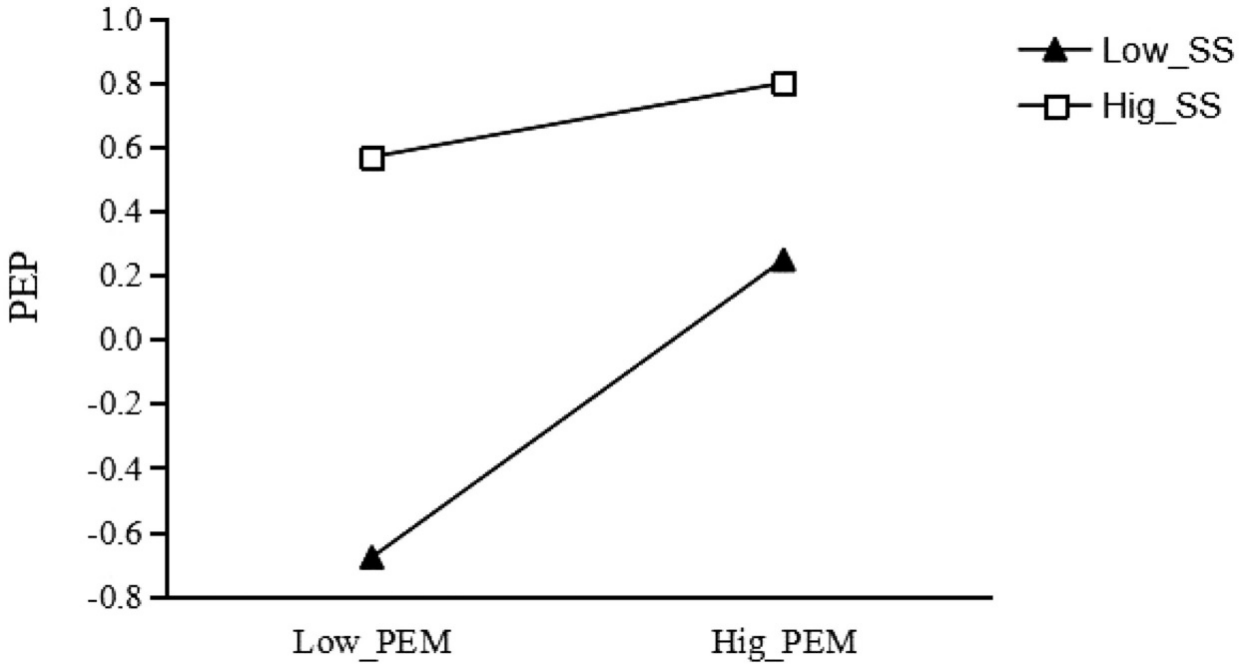
Moderating effect of social support on the relationship between exercise motivation and physical exercise persistence.

For the group with low social support (see Figure 5), the effect of exercise motivation on physical exercise persistence was significant (*β* = 0.463, *t* = 13.606, *p* < 0.001); for each unit increase in wearable sports equipment usage, college students' exercise motivation increased by 0.463 units. For the group with high social support, the effect of exercise motivation on physical exercise persistence remained significant (*β* = 0.116, *t* = 3.234, *p* < 0.01); for each unit increase in wearable sports equipment usage, college students' exercise motivation increased by 0.116 units. This indicates that social support plays a significant negative moderating role in the relationship between wearable sports equipment usage and exercise motivation.

## Discussion

This study, based on Self-Determination Theory (SDT) and Social Support Theory, explored the mechanisms by which wearable sports equipment influences college students' physical exercise persistence, focusing on the mediating role of exercise motivation and the moderating effect of social support. The findings not only validate the effectiveness of technological interventions in promoting college students' physical exercise behaviors but also reveal the dynamic role of social psychological factors in this process, providing both theoretical and practical guidance for the development of differentiated health promotion strategies.

### Direct impact of wearable sports equipment on physical exercise persistence

The study found that the use of wearable sports equipment significantly positively predicted college students’ physical exercise persistence, which is consistent with previous studies (20). According to The study found that the use of wearable sports equipment significantly positively predicted college students' physical exercise persistence, which is consistent with previous studies (20). According to Social Cognitive Theory (27), wearable devices help users build objective exercise cognitions by providing real-time feedback (such as steps, heart rate, and calories burned), thereby enhancing self-efficacy. For example, an intervention study showed that college students who used smart wristbands significantly increased their daily steps and improved self-regulation abilities (22). Additionally, the built-in achievement systems (e.g., badges, leaderboards) in devices reinforce exercise behaviors through operant conditioning (51), which aligns with the direct association between device usage and exercise persistence observed in this study. However, it is worth noting that relying solely on technological feedback may lead to “data fatigue” (38), where users experience burnout due to prolonged exposure to similar motivational patterns. This phenomenon suggests that the effects of technological interventions may be influenced by individual psychological factors (such as motivation levels) (17) and external environments (such as peer support) (46), providing a theoretical foundation for subsequent exploration of the roles of exercise motivation and social support.

### Mediating role of exercise motivation

The findings support the mediating role of exercise motivation in the relationship between wearable sports equipment usage and physical exercise persistence, which aligns with the core principles of Self-Determination Theory (17). Specifically, wearable sports equipment fosters the internalization of motivation by fulfilling the three basic psychological needs outlined by SDT—autonomy (setting personal goals), competence (demonstrating ability through data), and relatedness (social functions) (35). For example, empirical research that feedback on goal achievement provided by the equipment enhanced users' sense of competence, thereby increasing their intrinsic motivation (55). In studies with Chinese college students, evidence also showed that experimental groups using smart wristbands experienced a significant increase in exercise enjoyment (a key indicator of intrinsic motivation) (15).

However, the dynamic changes in motivation warrant attention. Longitudinal research indicates that initial equipment use relied on external motivation (such as earning virtual badges) to increase exercise frequency, but long-term persistence was significantly related only to intrinsic motivation levels (23). This finding resonates with the results of the moderating effect analysis in this study, where higher levels of social associated with a smaller promoting effect of exercise motivation on physical exercise persistence. This could be because, in high social support environments, individuals rely more on external incentives (e.g., peer encouragement) (52), whereas, in low social support environments, the role of intrinsic motivation is more pronounced (16).

### Moderating effect of social support

The moderating effect of social support in wearable sports equipment interventions presents a complex pattern. First, social support significantly enhanced the direct effect of wearable sports equipment usage on physical exercise persistence. At low levels of social support, the promoting effect of equipment usage was weaker, whereas at high levels of social support, this effect was significantly enhanced (Effect = 0.204, *p* < 0.001). This result is consistent with the Social Exchange Theory (46), which suggests that support from peers, family, or coaches can amplify the positive effects of technological interventions. For instance, empirical studies found that users who received offline guidance showed significantly higher exercise persistence compared to those using the equipment independently (22). In the Chinese cultural context, that family support significantly enhanced the positive impact of wearable equipment usage on exercise motivation (49). This may be because social support provides emotional validation and helps users internalize equipment feedback through behavioral modeling (47).

However, the moderating role of social support on exercise motivation presents a negative trend. At low levels of social support, the promoting effect of wearable sports equipment usage on exercise motivation was significant, but this effect was no longer significant at high levels of social support. This finding may be related to the “motivational crowding-out effect” (45), where external support (e.g., peer pressure) could undermine the autonomy of intrinsic motivation. Additionally, in high social support environments, users may rely more on interpersonal interactions rather than equipment feedback, leading to the weakening of the psychological mechanisms of the technological intervention (30). This result suggests that when formulating intervention strategies, it is important to balance the use of technology and social support, avoiding an overreliance on a single approach.

### Limitations of the study and future directions

Although this study provides important evidence for understanding the mechanisms behind the impact of wearable sports equipment on college students' physical exercise persistence, it still has certain limitations. First, this study employs a cross-sectional design to examine the immediate effects of wearable sports devices (WSE) on college students' exercise persistence. Given that temporal heterogeneity and long-term effects are crucial for technical intervention research, future studies could adopt longitudinal designs (e.g., baseline-3-month-12-month tracking) to further explore the sustained impacts of WSE usage and the evolution of motivation and social support. Additionally, subsequent research could integrate real-world device usage data (e.g., backend data from WSE devices) to investigate potential changes in “data fatigue”. Second, the sample only covered six universities in China and did not sufficiently consider differences in discipline, year of study, or regional variations. Future studies could expand the sample size to improve the external validity of the findings. Furthermore, the study did not differentiate between types of wearable sports equipment (e.g., smart wristbands vs. fitness watches) and their functional characteristics (e.g., feedback frequency, social interaction design). Future research could explore how different technical parameters affect the outcomes of interventions (26).

### Future research directions

Future studies could explore the following areas in greater depth: (1) Cross-cultural comparisons to analyze the moderating differences of social support in collectivist vs. individualistic cultures, building on recent frameworks for behavioral change interventions (45); (2) Integration of multimodal data, combining physiological indicators (such as heart rate variability) with behavioral data to construct more accurate predictive models, as demonstrated in recent athlete monitoring studies (13); (3) Optimization of technology pathways, designing dynamic incentive mechanisms based on behavioral economics (e.g., adaptive reward algorithms) to alleviate the issue of “data fatigue”, following principles from just-in-time adaptive interventions (31). These improvements will help promote the precise application of wearable sports equipment in the field of health promotion, particularly through personalized approaches identified in systematic reviews (43).

The findings of this study provide a theoretical foundation for sports intervention programs in higher education. Specifically, tailored WSE (Work-Study Engagement) interventions can be designed for students with varying levels of social support: Students with low social support could enhance their exercise persistence through personalized goals and virtual challenges, while those with high social support might further boost their motivation through group challenges and social interactions. Additionally, universities are advised to integrate WSE data with physical fitness test results to create dynamic health profiles, thereby supporting personalized intervention strategies.

## Conclusion

Based on self-determination theory and social support theory, this study explored the mechanisms through which wearable sports equipment influences college students' physical exercise persistence. The findings revealed that the use of wearable sports equipment significantly enhances college students' physical exercise persistence, with exercise motivation playing a partial mediating role. Social support exhibited a complex moderating effect within this model: on one hand, high levels of social support strengthened the direct promoting effect of equipment use on exercise persistence; on the other hand, social support weakened the positive relation between equipment use and exercise motivation and reduced the predictive relation between exercise motivation and physical exercise persistence. This suggests that for individuals with lower levels of social support, equipment use more significantly promotes exercise behavior indirectly by enhancing exercise motivation. In contrast, in high social support environments, the technological intervention provided by the equipment may partly replace the psychological drive of motivation. The results provide a theoretical basis for optimizing interventions in college students' physical exercise strategies, suggesting that differentiated social support plans be integrated with technological interventions to maximize the effect. Future research could further investigate the moderating differences from various sources of social support (e.g., peers, family, coaches) and examine the dynamic changes in long-term interventions.

## Data Availability

The raw data supporting the conclusions of this article will be made available by the authors, without undue reservation.

## References

[1] State Council of the People’s Republic of China. Healthy China 2030 planning outline (2016). Available online at: http://www.gov.cn/ (Accessed April 12, 2025).

[2] General Administration of Sport of China. Youth physical activity promotion plan (2017). Available online at: http://www.sport.gov.cn/ (Accessed April 12, 2025).

[3] State Council of the People’s Republic of China. National fitness plan (2021–2025) (2021). Available online at: http://www.gov.cn/ (Accessed April 13, 2025).

[4] Menhas R Yang L Saqib ZA Younas M Saeed MM. Does nature-based social prescription improve mental health outcomes? A systematic review and meta-analysis. Front Public Health. (2024) 12:1228271. 10.3389/fpubh.2024.1228271 38590811 PMC10999630

[5] Noor U Younas M Aldayel HS Menhas R Qingyu X. Learning behavior, digital platforms for learning and its impact on university student’s motivations and knowledge development. Front Psychol. (2022) 13:933974. 10.3389/fpsyg.2022.933974 36506979 PMC9726725

[6] Ministry of Education of the People’s Republic of China. Report on students’ physical fitness and health (2020). Available online at: http://www.moe.gov.cn/ (Accessed April 13, 2025).

[7] Bocarro JN Kanters MA Casper J Forrester S. School physical education, extracurricular sports, and lifelong active living. J Teach Phys Educ. (2008) 27(2):155–66. 10.1123/jtpe.27.2.155

[8] Geisen M Fox A Klatt S. VR as an innovative learning tool in sports education. Appl Sci. (2023) 13(4):2239. 10.3390/app13042239

[9] Cibrian FL Monteiro E Ankrah E Beltran JA Tavakoulnia A Schuck SEB Parents’ perspectives on a smartwatch intervention for children with ADHD: rapid deployment and feasibility evaluation of a pilot intervention to support distance learning during COVID-19. PLoS One. (2020) 15(10):e0241659. 34705845 10.1371/journal.pone.0258959 PMC8550607

[10] Di Fronso S Costa S Montesano C Di Gruttola F Ciofi EG Morgilli L The effects of COVID-19 pandemic on perceived stress and psychobiosocial states in Italian athletes. Int J Sport Exerc Psychol. (2022) 20(1):79–91. 10.1080/1612197X.2020.1802612

[11] Ministry of Education of the People’s Republic of China. Basic standards for physical education in higher education institutions (2014). Available online at: http://www.moe.gov.cn/ (Accessed April 13, 2025).

[12] Thompson WR Sallis R Joy E Jaworski CA Stuhr RM Trilk JL. Exercise is medicine. Am J Lifestyle Med. (2020) 14(5):511–23. 10.1177/1559827620912192 32922236 PMC7444006

[13] Ross R Arena R Myers J Kokkinos P Kaminsky LA. Update to the 2016 American Heart Association cardiorespiratory fitness statement. Prog Cardiovasc Dis. (2024) 83:10–5. 10.1016/j.pcad.2024.02.003 PMID: 38387825 38387825

[14] Woessner MN Tacey A Levinger-Limor A Parker AG Levinger P Levinger I. The evolution of technology and physical inactivity: the good, the bad, and the way forward. Front Public Health. (2021) 9:655491. 10.3389/fpubh.2021.655491 34123989 PMC8193221

[15] Jiang S Ng JY Choi SM Ha AS. Relationships among eHealth literacy, physical literacy, and physical activity in Chinese university students: cross-sectional study. J Med Internet Res. (2024) 26:e56386. 10.2196/56386 39496161 PMC11574492

[16] Milyavskaya M Koestner R. Psychological needs, motivation, and well-being: a test of self-determination theory across multiple domains. Pers Individ Dif. (2011) 50(3):387–91. 10.1016/j.paid.2010.10.029

[17] Ryan RM Deci EL. Self-Determination Theory: Basic Psychological Needs in Motivation, Development, and Wellness. New York, NY: Guilford Press (2017).

[18] Aroganam G Manivannan N Harrison D. Review on wearable technology sensors used in consumer sport applications. Sensors. (2019) 19(9):1983. 10.3390/s19091983 31035333 PMC6540270

[19] Brown CE Richardson K Halil-Pizzirani B Atkins L Yücel M Segrave A. Key influences on university students’ physical activity: a systematic review using the theoretical domains framework and the COM-B model of human behaviour. BMC Public Health. (2024) 24(1):418. 10.1186/s12889-023-17621-4 38336748 PMC10854129

[20] Fritz T Huang EM Murphy GC Zimmermann T. Persuasive technology in the real world: a study of long-term use of activity sensing devices for fitness. Proceedings of the SIGCHI Conference on Human Factors in Computing Systems (2014). p. 487–96

[21] Coulter R. Effect of wearable activity trackers and social media use on day-level physical activity motivation and behaviours (doctoral dissertation) (2023).

[22] Pope ZC Barr-Anderson DJ Lewis BA Pereira MA Gao Z. Use of wearable technology and social media to improve physical activity and dietary behaviors among college students: a 12-week randomized pilot study. Int J Environ Res Public Health. (2019) 16(19):3579. 10.3390/ijerph16193579 31557812 PMC6801802

[23] Steel RP. The longitudinal associations between wearable technology, physical activity and self-determined motivation. Int J Sport Exerc Psychol. (2024) 22(4):1031047. 10.1080/1612197X.2023.2180067

[24] Kononova A Li L Kamp K Bowen M Rikard RV Cotten S The use of wearable activity trackers among older adults: focus group study of tracker perceptions, motivators, and barriers in the maintenance stage of behavior change. JMIR Mhealth Uhealth. (2019) 7(4):e9832. 10.2196/mhealth.9832 30950807 PMC6473213

[25] Maher C Ryan J Ambrosi C Edney S. Users’ experiences of wearable activity trackers: a cross-sectional study. BMC Public Health. (2017) 17:1–8. 10.1186/s12889-017-4888-1 29141607 PMC5688726

[26] Shih PC Han K Poole ES Rosson MB Carroll JM. Use and adoption challenges of wearable activity trackers. Proceedings of the IConference 2015 (2015).

[27] Bandura A. Social Foundations of Thought and Action: A Social Cognitive Theory. Englewood Cliffs, NJ: Prentice Hall (1986).

[28] Tam KM. Application of the social cognitive theory to an electronic activity monitor system-based Physical Activity Intervention for working adults (doctoral dissertation). Baptist University, Hong Kong (2020).

[29] Bireline AM. The effect of wearable activity tracker social behaviors on moderate-to-vigorous physical activity and exercise self-efficacy (doctoral dissertation). The University of North Carolina at Greensboro (2024). 10.2196/75133 PMC13143198 42085670

[30] Hardeman W Houghton J Lane K Jones A Naughton F. A systematic review of just-in-time adaptive interventions (JITAIs) to promote physical activity. Int J Behav Nutr Phys Act. (2019) 16:1–21. 10.1186/s12966-019-0792-7 30943983 PMC6448257

[31] Nahum-Shani I Smith SN Spring BJ Collins LM Witkiewitz K Tewari A Just-in-time adaptive interventions (JITAIs) in mobile health: key components and design principles for behavior support. Ann Behav Med. (2018) 52(6):446–62. 10.1007/s12160-016-9830-8 27663578 PMC5364076

[32] Shin G Jarrahi MH Fei Y Karami A Gafinowitz N Byun A Wearable activity trackers, accuracy, adoption, acceptance and health impact: a systematic literature review. J Biomed Inform. (2019) 93:103153. 10.1016/j.jbi.2019.103153 30910623

[33] Peng X Menhas R Dai J Younas M. The COVID-19 pandemic and overall wellbeing: mediating role of virtual reality fitness for physical–psychological health and physical activity. Psychol Res Behav Manag. (2022) 15:1741–56. 10.2147/PRBM.S369020 35860203 PMC9289576

[34] Cairney J Dudley D Kwan M Bulten R Kriellaars D. Physical literacy, physical activity and health: toward an evidence-informed conceptual model. Sports Med. (2019) 49:371–83. 10.1007/s40279-019-01063-3 30747375

[35] Teixeira PJ Carraça EV Markland D Silva MN Ryan RM. Exercise, physical activity, and self-determination: a systematic review. Int J Behav Nutr Phys Act. (2012) 9(1):78. 10.1186/1479-5868-9-78 22726453 PMC3441783

[36] Ng JYY Ntoumanis N Thøgersen-Ntoumani C Deci EL Ryan RM Duda JL Self-determination theory applied to health contexts: a meta-analysis. Perspect Psychol Sci. (2012) 7(4):325–40. 10.1177/1745691612447309 26168470

[37] Peng W Lin J-H Crouse J. Is playing exergames really exercising? A meta-analysis of energy expenditure in active video games. Cyberpsychol Behav Soc Netw. (2016) 14(11):681–8. 10.1089/cyber.2010.0578 21668370

[38] Attig C Franke T. Why do people abandon activity trackers? The role of user diversity in discontinued use. Int J Hum Comput Interact. (2023) 39(8):1662–74. 10.1080/10447318.2022.2067935

[39] Lazar A Koehler C Tanenbaum J Nguyen DH. Why we use and abandon smart devices. Proceedings of the 2015 ACM International Joint Conference on Pervasive and Ubiquitous Computing (2015). p. 635–46

[40] Younas M Dong Y Menhas R Li X Wang Y Noor U. Alleviating the effects of the COVID-19 pandemic on the physical, psychological health, and wellbeing of students: coping behavior as a mediator. Psychol Res Behav Manag. (2023) 16:5255–77. 10.2147/PRBM.S441395 38164325 PMC10758179

[41] Au WW Recchia F Fong DY Wong SHS Chan DKC Capio CM Effect of wearable activity trackers on physical activity in children and adolescents: a systematic review and meta-analysis. Lancet Digit Health. (2024) 6(9):e625–39. 10.1016/S2589-7500(24)00139-0 PMID: 39112110 39112110

[42] Casado-Robles C Viciana J Guijarro-Romero S Mayorga-Vega D. Effects of consumer-wearable activity tracker-based programs on objectively measured daily physical activity and sedentary behavior among school-aged children: a systematic review and meta-analysis. Sports Med Open. (2022) 8(1):18. 10.1186/s40798-021-00407-6 35099630 PMC8804065

[43] Ferguson T Olds T Curtis R Blake H Crozier AJ Dankiw K Effectiveness of wearable activity trackers to increase physical activity and improve health: a systematic review of systematic reviews and meta-analyses. Lancet Digit Health. (2022) 4(8):e615–26. 10.1016/S2589-7500(22)00111-X 35868813

[44] Tang MSS Moore K McGavigan A Clark RA Ganesan AN. Effectiveness of wearable trackers on physical activity in healthy adults: systematic review and meta-analysis of randomized controlled trials. JMIR Mhealth Uhealth. (2020) 8(7):e15576. 10.2196/15576 32706685 PMC7407266

[45] Albarracín D Fayaz-Farkhad B Granados Samayoa JA. Determinants of behaviour and their efficacy as targets of behavioural change interventions. Nat Rev Psychol. (2024) 3(6):377–92. 10.1038/s44159-024-00305-0 40909436 PMC12407158

[46] Hunter RF de la Haye K Murray JM Badham J Valente TW Clarke M Social network interventions for health behaviours and outcomes: a systematic review and meta-analysis. PLoS Med. (2019) 16(9):e1002890. 10.1371/journal.pmed.1002890 31479454 PMC6719831

[47] Short CE DeSmet A Woods C Williams SL Maher C Middelweerd A Measuring engagement in eHealth and mHealth behavior change interventions: viewpoint of methodologies. J Med Internet Res. (2018) 20(11):e292. 10.2196/jmir.9397 30446482 PMC6269627

[48] Xu Y Peng J Jing F Ren H. From wearables to performance: how acceptance of IoT devices influences physical education results in college students. Sci Rep. (2024) 14(1):23776. 10.1038/s41598-024-75071-3 39390061 PMC11467443

[49] Sun M Jiang LC. Linking social features of fitness apps with physical activity among Chinese users: evidence from self-reported and self-tracked behavioral data. Inf Process Manag. (2022) 59(6):103096. 10.1016/j.ipm.2022.103096

[50] Rieger E Sellbom M Murray K Caterson I. Measuring social support for healthy eating and physical activity in obesity. Br J Health Psychol. (2018) 23(4):1021–39. 10.1111/bjhp.12336 30054957

[51] Song J Kim J Cho K. Understanding users’ continuance intentions to use smart-connected sports products. Sport Manag Rev. (2018) 21(5):477–90. 10.1016/j.smr.2017.10.004

[52] Wang S Liu YP Gu CQ. Effects of team cohesion on exercise adherence in amateur sports: a moderated two-level mediation model. J Wuhan Univ Phys Educ. (2016) 50:73–80+85. 10.15930/j.cnki.wtxb.2016.03.012

[53] Vaux A Phillips J Holly L Thomson B Williams D Stewart D. The social support appraisals (SS-A) scale: studies of reliability and validity. Am J Community Psychol. (1986) 14(2):195–219. 10.1007/BF00911821

[54] Luo XF Chen QS Mu SK. Chinese revision and preliminary application of the Child and Adolescent Social Support Scale (CASSS). Chin J Clin Psychol. (2017) 25(4):671–4.

[55] Rupp MA Michaelis JR McConnell DS Smither JA. The role of individual differences on perceptions of wearable fitness device trust, usability, and motivational impact. Appl Ergon. (2018) 70:77–87. 10.1016/j.apergo.2018.02.005 29866329

